# Supplementary figures and images for: Klebsiella pneumoniae OmpR facilitates lung infection through transcriptional regulation of key virulence factors
Source: Microbiol Spectr. 2023 Dec 15;12(1):e03966-23. doi: 10.1128/spectrum.03966-23 (PMC10783089; doi:10.1128/spectrum.03966-23)

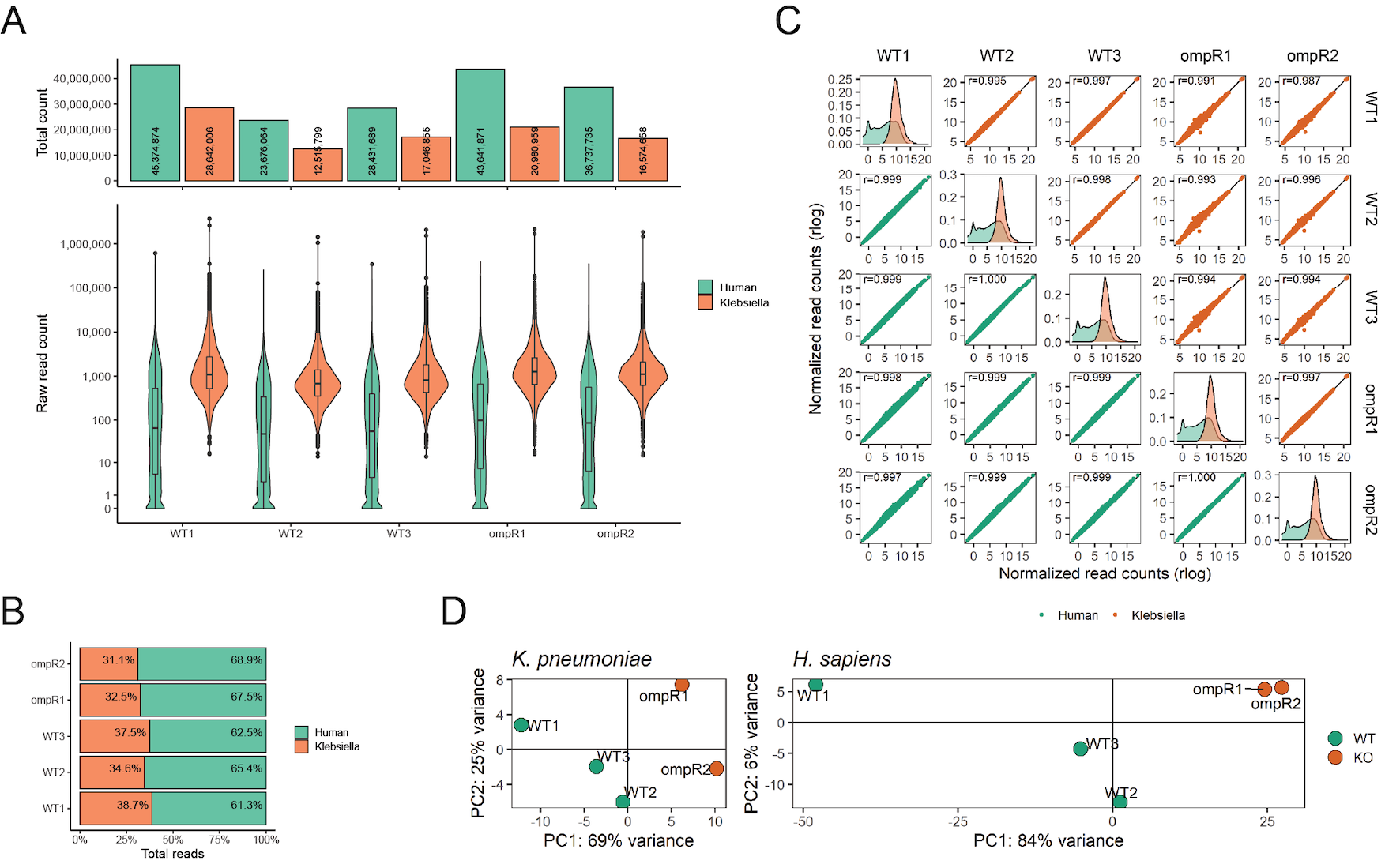

Supplement: Figure S1 — Composition and mapping of dual RNA-seq Illumina sequencing reads. [file spectrum.03966-23-s0001.tiff]

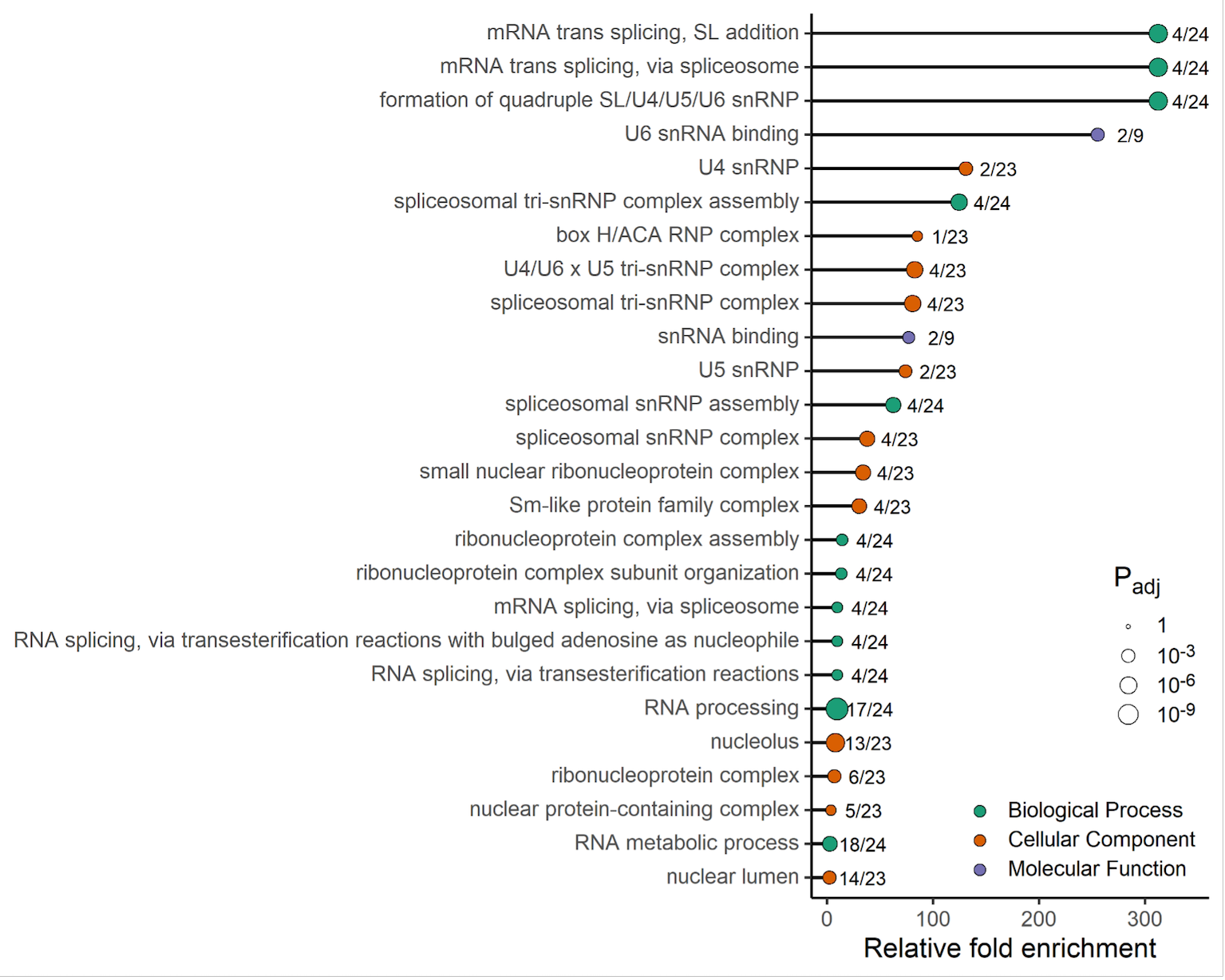

Supplement: Figure S2 — GO term enrichment analysis of significantly downregulated genes in lung epithelial cells in response to ΔompR K. pneumoniae. [file spectrum.03966-23-s0002.tiff]
