# Supplementary material for: Klebsiella pneumoniae OmpR facilitates lung infection through transcriptional regulation of key virulence factors
Source: Microbiol Spectr. 2023 Dec 15;12(1):e03966-23. doi: 10.1128/spectrum.03966-23 (PMC10783089; doi:10.1128/spectrum.03966-23)
Supplement: Supplemental legends — Legends for supplemental Figures S1 and S2. [file spectrum.03966-23-s0003.docx]

# Supplementary Figure Legends

## Supplementary Figure S1

**Composition and mapping of dual RNA‑seq Illumina sequencing reads.** **A)** Distribution of the aligned and counted sequencing reads between the five libraries, split out per organism. The samples had an average of 54.7 million reads (range: 36.2 to 74.0 million reads) per library. **B)** The proportion of the sequencing reads of each sample mapped to the *K. pneumoniae* ATCC 43816 genome, and the human genome. **C)** Intra‑library distributions and inter‑library correlations of normalized counts. Read counts were normalized with a blind rlog transformation using DESeq2. **D, E)** Principal component analyses (PCA) plots of read counts for *K. pneumoniae* (panel D) and *Homo sapiens* (panel E).

## Supplementary Figure S2

**GO term enrichment analysis of significantly downregulated genes in lung epithelial cells in response to Δ*ompR K. pneumoniae*.** Relative fold enrichment is the gene ratio (faction of downregulated genes in GO gene set divided by downregulated genes in GO data base) divided by the background ratio (fraction of total genes in GO set divided by total genes in GO data base). Ratios displayed in plot are gene ratios. All significantly enriched GO terms are shown (Padj<0.05). Complete table of results can be found as Supplementary Table S5.
